# Impact of nanoparticles on the *Bacillus subtilis* (3610) competence

**DOI:** 10.1038/s41598-018-21402-0

**Published:** 2018-02-14

**Authors:** Elise Eymard-Vernain, Sylvie Luche, Thierry Rabilloud, Cécile Lelong

**Affiliations:** Université Grenoble Alpes, CNRS, CEA, BIG, CBM, 17 avenue des Martyrs, 38054 Grenoble cedex 9, France

## Abstract

Due to the physicochemical properties of nanoparticles, the use of nanomaterials increases every year in industrial and medical processes. At the same time, the increasing number of bacteria becoming resistant to many antibiotics, mostly by a horizontal gene transfer process, is a major public health concern. We herein report, for the first time, the role of nanoparticles in the physiological induction of horizontal gene transfer in bacteria. Besides the most well-known impacts of nanoparticles on bacteria, i.e. death or oxidative stress, two nanoparticles, n-ZnO and n-TiO_2_, significantly and oppositely impact the transformation efficiency of Bacillus subtilis in biofilm growth conditions, by modification of the physiological processes involved in the induction of competence, the first step of transformation. This effect is the consequence of a physiological adaptation rather than a physical cell injury: two oligopeptide ABC transporters, OppABCDF and AppDFABC, are differentially expressed in response to nanoparticles. Interestingly, a third tested nanoparticle, n-Ag, has no significant effect on competence in our experimental conditions. Overall, these results show that nanoparticles, by altering bacterial physiology and especially competence, may have profound influences in unsuspected areas, such as the dissemination of antibiotic resistance in bacteria.

## Introduction

Due to the specific physicochemical properties of nanoparticles, e.g. high specific area, the use of nanomaterials increases every year in industrial and technological processes or in medical applications. Despite their exceptional qualities, their deleterious impact on the environment and health also gives rise to an increasing number of publications^1–4^. Among their numerous uses, nanoparticles and particularly metal-oxide nanoparticles, have often been used for their antibacterial properties, reviewed in^5,6^. They are also considered an alternative to antibiotic treatment because they can efficiently kill bacteria with no or a very limited emergence of drug resistance to date. Most often, the deleterious impacts of nanoparticles on cells and especially on bacteria are described as the result of a physical injury to cell integrity, either by disruption of the membrane and/or by the production of reactive oxygen species^5,7^. Some recent publications have shown that nanoparticles can also impact internal physiological processes of bacteria such as stringent response, respiration, central metabolism, motility, sporulation or chromosome condensation^8–10^. We have shown that exposure of the soil bacterium *Bacillus subtilis* (laboratory strain, 168) to n-TiO_2_ and n-ZnO, under long-term adaptation growth conditions in a liquid medium, impacts its physiological state by modifying the central metabolism and stringent response^8^. However, the natural biotope of *Bacillus subtilis* is the upper layer of soil, where it grows as a biofilm. It plays a role in rhizospheric processes as a symbiotic organism for plant^11^. To mimic this physiological development and study the impact of nanoparticles during the formation of a biofilm in a contaminated soil, we studied the proteomic response of the ancestral strain *Bacillus subtilis* 3610, which is able to form a biofilm, contrary to the well known 168 laboratory strain. The bacteria were grown on soft agar plates containing n-ZnO and n-TiO_2_. We show here that under these growth conditions, where the nanoparticles are not physically in contact with bacteria, n-TiO_2_ and n-ZnO impact, in an opposite way, the expression of two oligopeptide ABC transporters, OppABCDF and AppABCDF, and consequently, the competence of *Bacillus subtilis*. The competence being one of first step of the transformation process, our results suggest that nanoparticles impact horizontal gene transfer in, at least Gram positive bacteria and may have significant impact on the appearance of antibiotic-resistant bacteria.

## Results

### Nanoparticles characterization

The sizes and aggregation states of the nanoparticles of n-TiO_2_ and n-ZnO suspensions were characterized by DLS measurements^8^ and TEM images analysis (sup data 1). The nominal size and morphological characteristics of n-TiO_2_ and n-ZnO were observed by transmission electron microscopy in H_2_O suspension. Supp data 1 shows representative images of each. The measured nominal sizes were 17 nm ± 7.4 and 20.3 nm ± 7.3 for n-TiO_2_ and n-ZnO, respectively. In Luche *et al*.^8^, the measured diameter using DLS approaches were 70 nm ± 45 and 139 nm ± 21. These measurements were representative of the aggregations observed on microscopy images (Supp data 1). The n-Ag were extensively characterized by Eymard *et al*.^12^ in liquid LB medium. In this work, the nanoparticles were very rapidly embedded in the agar LB gelose after their sequential dilution in H_2_O. Moreover, we could not detect significant amounts of TiO_2_ (by ICP-AES measurements), neither in the intracellular extract nor in the swarming bacterial colony (data not shown). The bacteria in the swarming colony have also been observed by electron microscopy (data not shown), and no nanoparticles, neither TiO_2_ nor ZnO, have been detected. Thus, we postulated that the nanoparticles keep similar characteristics in the agar medium than in water suspension and were not directly in contact with the bacteria cells during the growth on agar medium.

### Proteomic analysis

**Figure 1:**
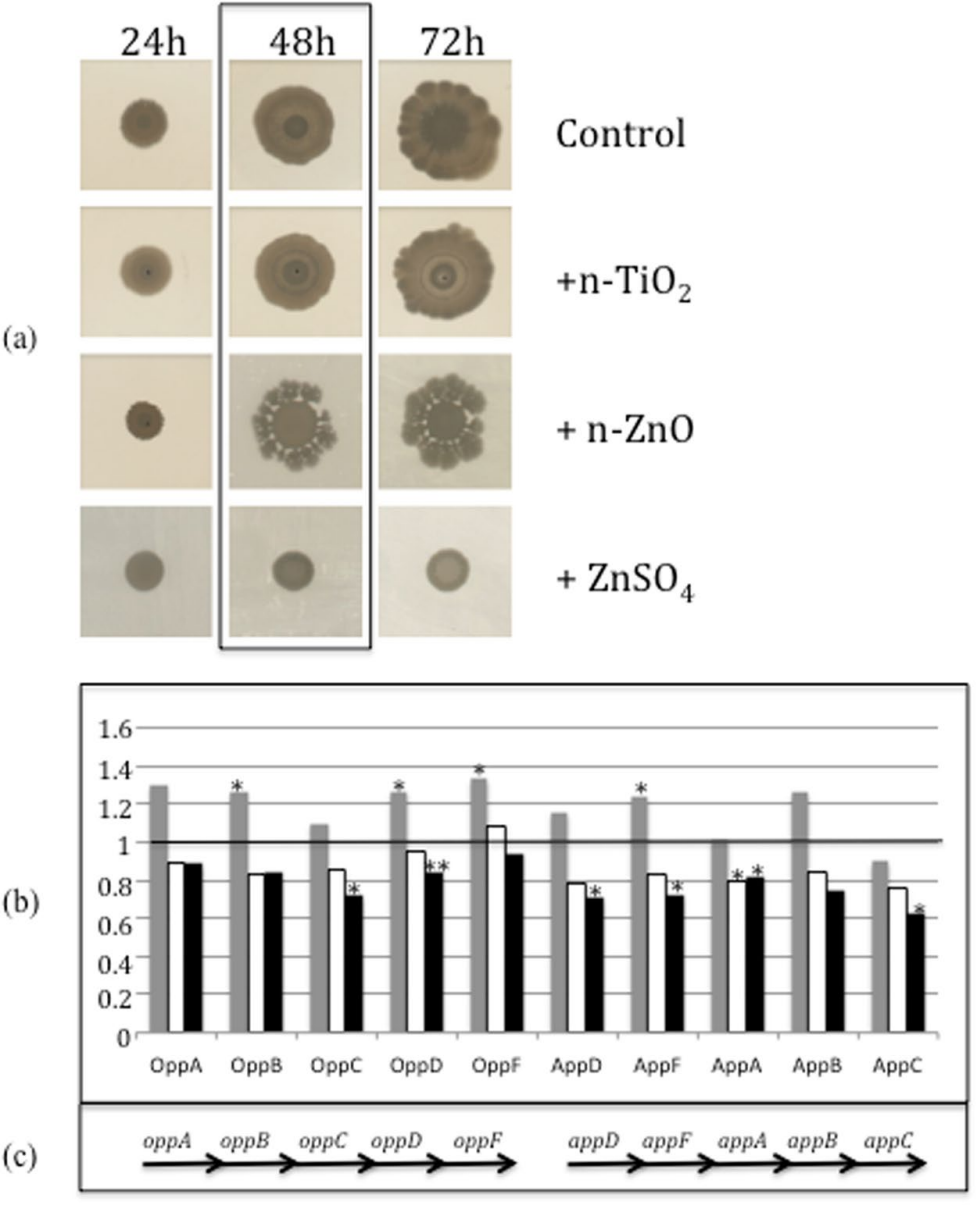
(**a**) Effect of nanoparticles on *Bacillus subtilis* growth. Colony biofilms formed on LB soft agar containing no nanoparticle, n-TiO_2_, n-ZnO or ZnSO_4_ salt after 24, 48 and 72 h of growth at 37 °C. (**b**) Expression ratio of the OppABCDF and AppDFABC proteins determined by proteomic analysis: Compared to the control: in grey, ratio of the protein expression in presence of n-TiO_2_/protein expression in the control growth condition, in white, in presence of n-ZnO and in black, in presence of ZnSO_4_. (**c**) Schematic representation of the operons coding for both oligopeptides ABC transporter. Asterix * and **indicate significant differences *p* < 0.05 and *p* < 0.005, respectively.

The 3610 *Bacillus subtilis* strain was grown on a soft agar LB plate to promote the swarming motion during the formation of biofilm. Figure 1 shows the macroscopic phenotypes from 24 to 72 h of growth on soft agar containing nanoparticles: the growth was similar with or without n-TiO_2_ nanoparticles. In contrast, the growth seems to be slowed down and stopped after 24 h in the presence of n-ZnO and ZnSO_4_ salt, respectively. The cells were collected after 48 h of growth to be analysed by shotgun proteomics, and the data are available at the PRIDE repository (data set identifier PXD006444). In view of the very different macroscopic phenotypes of the biofilms observed in response to n-TiO_2_ and n-ZnO or ZnSO_4_ (Fig. 1a), we decided to focus our analysis on proteins for which the proteomic data showed an opposite expression profile when we compared, on one hand, the direction of the variation of n-ZnO or ZnSO_4_/control and, on the other hand, the variation of n-TiO_2_/control (Fig. 1b). Among all of the proteins showing a significant modification in their expression, ten proteins were highlighted: OppA, OppB, OppC, OppD, OppF, AppA, AppB, AppC, AppD and AppF, which are encoded by two operons, *oppABCDF* and *appDFABC*, respectively (Fig. 1c). All of the proteins coded by both operons have been detected by proteomic analysis. The fold change between control and the exposed cells was always low, from 1.1 to 1.4 for an increase and from 0.7 to 0.95 for a decrease. However, their expression profiles were consistently modified in the same way in both operons: they were all increased in the presence of n-TiO_2_ and decreased in the presence of n-ZnO or ZnSO_4_ salt (Fig. 1b), with only two exceptions, OppF and AppC for the ratio n-ZnO/control and n-TiO_2_/control, respectively, for which the t-test were not significant. Both operons code for the same functional multimer protein, an oligopeptide ABC transporter^13–16^. The App transporter can completely substitute Opp transporter in two main cellular mechanisms: the sporulation and the competence^14,16–19^. Mutations and deletion in the Opp operon affect the specifity of oligopeptides transport but the effect on competence were dependent of the mutation: partly^19,17^ or completely defective^16^. Zaprasis and collaborators^20^ have also described the involvement of Opp and App transporters in the osmoprotection of the bacteria by the import of proline-containing peptides. Their transcriptional expression is dependent on the growth phase: the transcription of *opp* occurs during the exponential growth, while *app* is expressed during the stationary phase^18^. In our conditions, both operons show the same protein expression profile, which is not correlated to the growth phase but to the response to an external signal produced directly or indirectly by the presence of the nanoparticles and salts. We decided to explore the effect of nanoparticles on the three cellular mechanisms known to be linked to the Opp and App ABC transporters: osmoprotection, sporulation and competence, mechanisms which have been described in the literature to be linked to the both ABC transporters^13–16,20^.

### Role of the Opp and App ABC transporters in response to nanoparticles

#### The osmotic stress controlled by the import of proline-containing peptides

**Figure 2:**
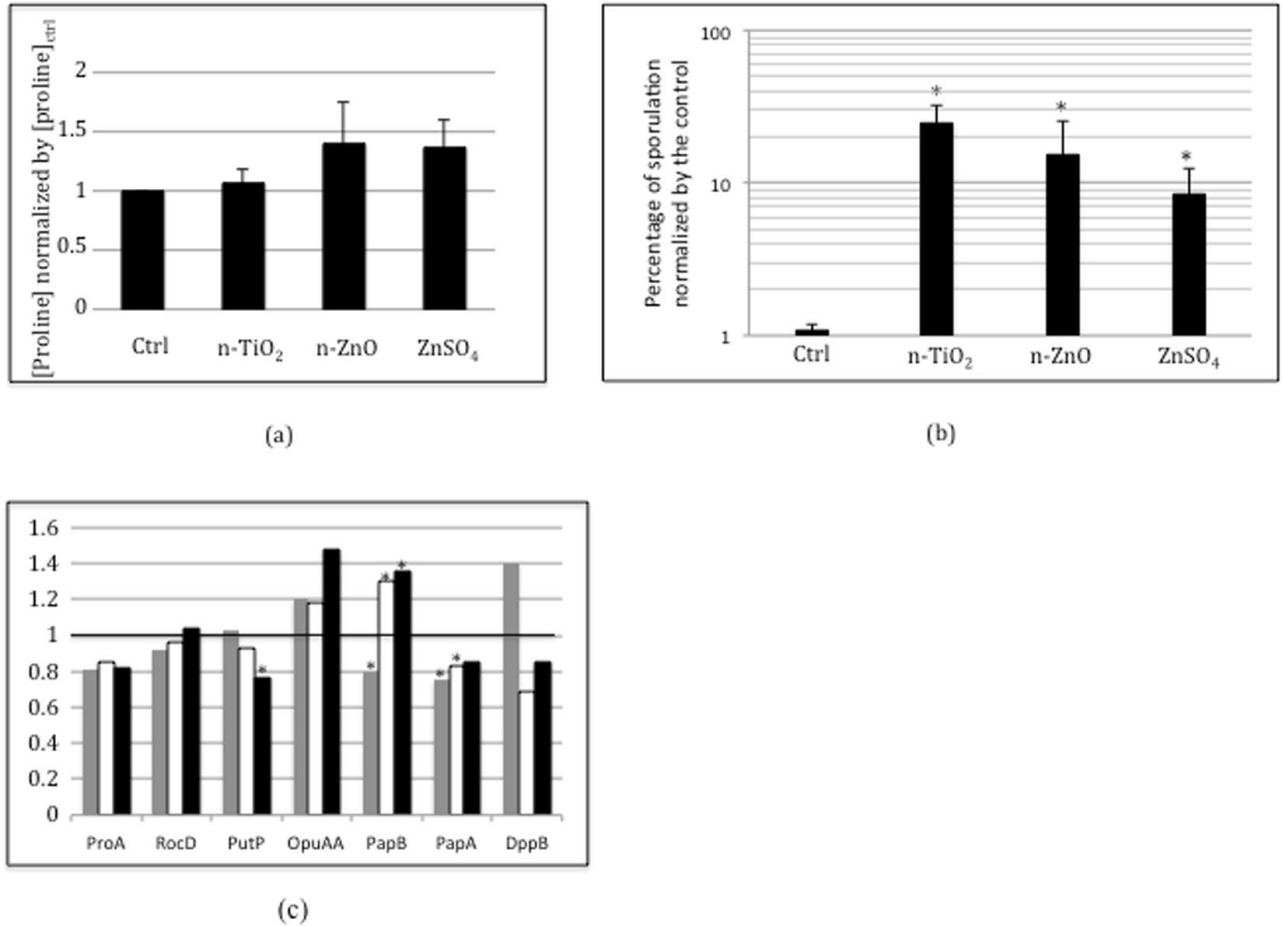
Effect of nanoparticles on the [proline]_intracellular_ and the sporulation (**a**) Proline assay: [Proline] was normalised by the [proline] measured in the control (**b**) Percentage of sporulation = (number of spore forming/CFU) × 100. Error bars ± 1 SD (*n* ≥ 3). All differences are significant with *p* < 0.05. (**c**) Expression ratio of the ProA, the glutamate-5-semialdehyde dehydrogenase, RocD, the ornithine transaminase, PutP, responsible for the proline uptake, OpuAA, the glycine betaine ABC transporter, PapA, an Xaa-Pro amino-peptidase, and its paralogue PapB and DppB, a dipeptide transorter permease, proteins determined by proteomic analysis: in grey, ratio of the protein expression in presence of n-TiO_2_/normalized by the protein expression in the control growth condition, in white, ratio of the protein expression in presence of n-ZnO/normalized by the protein expression in the control growth condition and in black, ratio of the protein expression in presence of ZnSO_4_/normalized by the protein expression in the control growth condition. Asterix indicates significant differences *p* < 0.05.

To determine whether the nanoparticles and salts have been sensed as an osmotic stress controlled by the import of proline-containing peptides, which is mediated by the App and Opp transporters, we measured the intracellular proline concentration. No significant differences were observed after 48 h of growth on soft agar, with or without nanoparticles or salt (Fig. 2a). Moreover, except Dpp, a dipeptide transorter permease, no other protein known to be involved in this type of osmotic response^20–23^, such as OpuAA, the glycine betaine ABC transporter, PutP, responsible for the proline uptake, PapA, an Xaa-Pro amino-peptidase, or its paralogue PapB, showed a similar expression profile to those of the Opp and App transporters (Fig. 2b). The expression profile of Opp and App, in the presence of nanoparticles or salt, is not the result of osmotic stress controlled by the import of proline-containing peptides.

#### The sporulation

Then, we compared the percentage of sporulation (=number of spores/total CFU) × 100) after the exposition to nanoparticles. In all treated cells, we observed an increase in sporulation in the same order of magnitude, of around ten (Fig. 2c), compared to the control condition, which cannot be correlated with the differential expression profile of Opp and App transporters observed in the presence of nanoparticles (Fig. 1b). The sporulation process is under the control of a complex regulatory network reviewed in^23^. It should be noted that the proteomic analysis has allowed the identification of proteins specific to the sporulation state but no significant difference concerning their expression has been detected (PRIDE repository PXD006444). Thus, the differential expression of the Opp and App transporters is not directly linked to the sporulation process.

#### Competence

**Figure 3:**
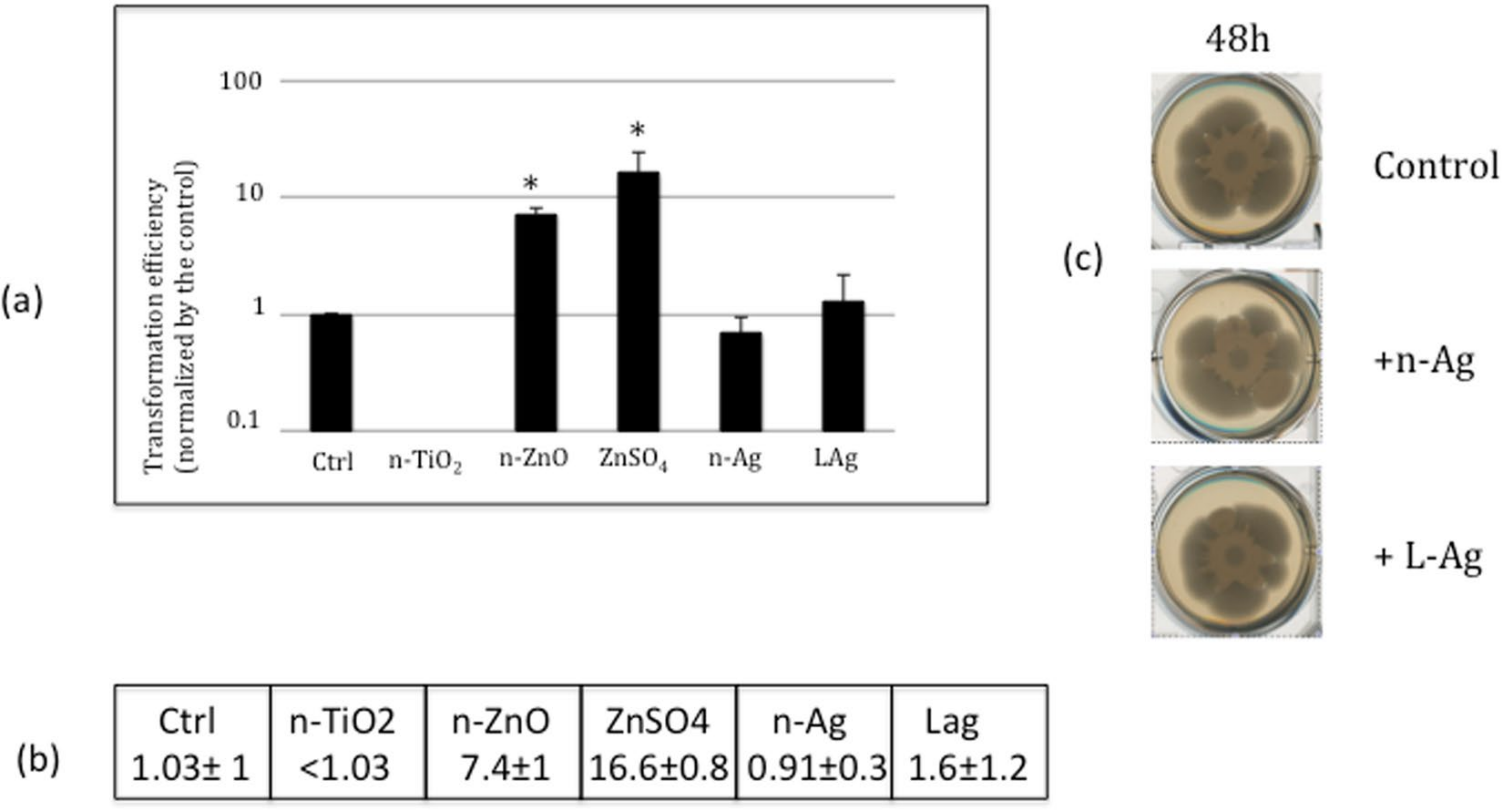
Effect of nanoparticles on the *Bacillus subtilis* competence. The cell exposed to nanoparticles, n-TiO_2_, n-ZnO or n-Ag or salts, ZnSO_4_ or silver lactate, during 48 h of growth on soft agar were recovered and incubated with the pJim plasmid. The transformation efficiency was measured using a replicative plasmid pJim as described in the methods. The presence of the plasmid pJim in cells resistant to erythromycin was detected by PCR analysis using primers specific to the pJim plasmid. All erythromycin resistant colonies contained the pJim plasmid (data not shown). (**a**) Transformation efficiency after exposition to nanoparticles or salt normalized by transformation efficiency in the control growth condition. Error bars ± 1 SD (*n* ≥ 3). Asterix indicates significant differences *p *< 0.05. (**b**) Transformation efficiency (transformants/CFU): Number of erythromycin resistant colonies/10^9^ colony forming unit. (**c**) Colony biofilms formed on LB soft agar containing no nanoparticle, n-Ag or Silver Lactate after 48 of growth at 37 °C.

**Figure 4:**
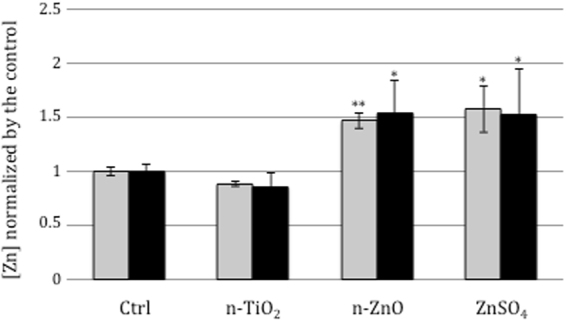
Intracellular Zn concentration/mg protein. All the assays were performed in triplicate and at least two technical replicates. In grey, [Zn]_intracellular_ and in black, [Zn]_total_. The results were normalized by the value obtained in the control condition. Error bars ± 1 SD (*n* ≥ 3). Asterix * and ** indicate significant differences *p* < 0.05 and *p* < 0.005, respectively.

The expression profile of the Opp and App ABC transporters in the presence of n-TiO_2_ and n-ZnO and ZnSO_4_ salt was not correlated to a modification of the sporulation or a response to osmotic stress by the import of proline-containing peptides; therefore, we tested the third mechanism linked to both ABC transporters, the competence of bacteria, i.e. their ability to bring DNA into the cells, which is the first step of transformation, one of the mechanisms involved in horizontal gene transfer. We measured the transformation efficiency using a replicative plasmid pJim, after 48 h of growth on soft agar containing nanoparticles or salt, as described in the methods. The presence of the plasmid pJim in cells resistant to erythromycin was detected by PCR analysis using primers specific to the pJim plasmid. All erythromycin resistant colonies contained the pJim plasmid (data not shown). Figure 3a,b shows that the transformation efficiency was significantly affected after exposure of the cells to nanoparticles or salt: under the control conditions, we observed a transformation frequency (transformants/CFU) of around 10^−9^, whereas in the presence of n-TiO_2_, we were never able to observe a transformant cell. Also in the presence of n-ZnO and ZnSO_4_, we observed a 10-fold increase in the transformation frequency, of around 10^−8^. Despite the fact that biofilm growth conditions are clearly not optimised to trigger the competent process, we have observed significant and opposite effects of the nanoparticles n-TiO_2_ and n-ZnO on the competence. The effect was the same with n-ZnO and ZnSO_4_ salt: an increase of the competence with the same magnitude order. It has already been described that in rich liquid bacteria mediun, n-ZnO is dissolved in Zn^2+^ ions and complexed with amino acids contained in the medium^24^. If we consider total dissolution, the soft agar plates would contain 0.2 mM and 0.03 mM of Zn^2+^ ions with n-ZnO and ZnSO_4_, respectively. Under both conditions, the amino acid and peptide concentrations provided by the tryptone and yeast extract in the LB medium were quite sufficient to complex all of the Zn^2+^ ions. The fact that similar phenotypes, macroscopic growth (Fig. 1a), Opp and App transporter expression profiles (Fig. 1b,c) and competence (Fig. 3a,b), were observed with n-ZnO and ZnSO_4_ salt drove us to hypothesise that only a portion of n-ZnO was effectively dissolved in soft agar medium. This may correspond to≤0.03 mM dissolved Zinc, which corresponds to≤15% of n-ZnO dissolved in the agar medium. This hypothesis was confirmed by the total absence of growth on soft agar plates containing 0.3 mM of ZnSO_4_ salt (data not shown) and moreover, by the ICP assays, which have shown that [Zn]_intracellular_ was significantly increased but with the same magnitude order, in both conditions while the potential total quantity of Zn ions is ten times higher with n-ZnO than with ZnSO_4_ (Fig. 4). The Zn accumulation has also been measured in all the swarming colony, i.e. [Zn]_total_, which includes the intracellular content and also the exopolymeric substances secreted in the matrix surrounding the bacteria cells in the swarming. As Fig. 4 shown, the increasing of the [Zn]_total_ was identical to the increasing of the [Zn]_intracellular_. Because of the absence of physical contact between nanoparticles and bacteria in our conditions and the homology of phenotypes, we concluded that the observed phenotypes were due to the ≈15% of dissolved Zn^2+^ ions.

To test the specific role of nanoparticles in the variation of the competence of a bacterial cell, we have measured the transformation efficiency in the presence of a third nanoparticle which is also known to have a high level of dissolution in growth culture medium: n-Ag. As for the exposure with n-TiO_2_ and n-ZnO, the bacteria were exposed to 1-ppm n-Ag or Silver lactate embedded in soft agar for 48 h. In all conditions, the biofilm has the same macroscopic phenotype (Fig. 3c). The cell exposed to n-Ag or silver lactate were recovered and incubated with the pJim plasmid. Figure 2a,b shows that in the presence of n-Ag or silver lactate as an ionic control, no significant difference in the transformation efficiency was observed.

## Discussion

While the Opp and App transporters involved in signal transduction pathways regulating bacterial development, virulence or competence in different bacteria^25–30^, in *Bacillus subtili,s* they are involved in three main pathways: the osmoregulation, the sporulation and the competence^14–16,18,20,22^. In presence of nanoparticles, the proteome analysis has shown a new and unexpected protein profile expression of these both ABC transporter: an increasing in presence of TiO_2_ and a decreasing in presence of n-ZnO or ZnSO_4_. This protein profile expression was not correlated to a response of *Bacillus subtilis* to nanoparticles by the induction of sporulation or the osmoregulation response. It was inversely correlated to the induction of competence: while n-TiO_2_ inhibits and n-ZnO increases the *Bacillus subtilis* competence, a third nanoparticle, n-Ag, has no effect. The competence process from its induction to the complete entry of DNA in the cell is very complex and not totally understood: the Opp and App transporters are responsible for the import of the extracellular peptide factors, which initiate the competence process. The complete process involves many regulation cascades, transporters, sensors and enzymes and the situation is probably even more complex in the context of a biofilm formation^31–34^. The population of a biofilm is extremely heterogeneous in its architecture and cell composition^35^. In a biofilm, *Bacillus subtilis* can be differentiated in different communities, considering their main physiological pathway, that coexists in the same environment: motile cells, spores, cannibal cells, matrix producers, competent, surfactin producers, dead cells or undifferentiated cells^35,36^. In the literature^17,19^, the deletion or mutation of Opp leads to a decrease of the competence and the sporulation and their expression are correlated to the growth phase^18^. In a biofilm on soft agar, we have observed a systematic increasing of the sporulation, whatever the applied stress (see Supp data 2 for the silver exposition) and an opposite phenotype concerning the competence, i.e. the expression profile of Opp and App is inversely correlated to the transformation efficiency in presence of n-TiO_2_ and n-ZnO, and the same expression profile for the both ABC transporter. The protein expression profile reflects the proportion of each protein accumulated during 48 h in a complex and heterogeneous mix of cells, the transformation efficiency only reflects the ability of some categories of bacteria, physiologically active and able to become competent. In any case, our results highlight an unknown and new regulatory pattern of the competence pattern or at least of the Opp/App expression in response to nanoparticles.

The transformation frequency measured in the control conditions is far lower than the values reported in the literature, even for the 3610 strain^37–39^, where it is less than 10^−8^ to 10^−6^. However, in all of these publications, the authors have optimised the competence of the cells by conditioning them with growth conditions known to induce the competent process (e.g. deficiency in amino acids) or by genetic modifications (curing the pBS32 plasmid^38^). It is well documented that even under favourable conditions, only a small proportion of the population induces the cascade of gene expression required for the competent phenotype. However, low transformability seems to be standard for wild-type *Bacillus subtilis* strains, such as the 3610 strain, but the ecological parameters favouring genetic competency remain largely obscure, as described by Jakobs and Meinhardt^34^. It has to be noticed that several publications have already shown that nanoparticles may be involved in a better transformation efficiency^40–45^. But, in all theses cases, the authors demonstrated that it was due to a mechanical effect of the nanoparticles: protection of the DNA from DNase activity by interaction between DNA and nanoparticles^41^ or damage to the cell envelope which improves the DNA uptake^43–45^, i.e. phenomena that occur only under laboratory growth conditions in an agitated liquid medium. In our case, where the nanoparticles were embedded in the agar and have at most a very limited direct contact with the bacteria, the proteomic data show that nanoparticles have significant impacts on the physiology of bacteria by indirect mechanisms that are still unclear. One physiological consequence is modification of their competence and, consequently, their ability to acquire new characteristics by horizontal gene transfer.

In the presence of n-ZnO, the modification of competence is probably the result of a partial dissolution of the nanoparticles into Zn ions. Li and colleagues have shown, on the one hand, that n-ZnO is completely dissolved into Zn^2+^ ions in liquid LB medium and, on the other hand, that most Zn^2+^ ions are complexed with amino acids resulting in a dramatic decrease of the n-ZnO toxicity^24^. In our conditions, where n-ZnO is embedded in agar plate, the results let suppose that n-ZnO in a solid and complex biological medium is not totally dissolved and moreover, may act as sustained delivery zinc storage with a long-term toxicological impact.

We have demonstrated that, besides the most known impacts of nanoparticles on bacteria, i.e. death or the induction of oxidative stress, the nanoparticles also modify the competence of the Gram positive bacteria, *Bacillus subtilis*. This modification has been observed in growth conditions corresponding to a long-term adaptation to nanoparticles and at concentrations close to those observed in the natural environment (≤1ppm)^46^. In the case of n-ZnO and n-Ag, which are known to be largely dissolved in the corresponding ion in liquid biological media, the physiological effects were completely different: an increase of competence and no impact, respectively. In contrast, after exposure to n-TiO_2_, the competence was significantly decreased. Under the same growth conditions, long-time adaptation and biofilm formation on soft agar, the exposure to nanoparticles has given rise to very different and opposite phenotypes depending on the nanoparticle considered.

DNA uptake, followed by the recombination and/or the replication, is a natural way for bacteria to survive and adapt their genome to environmental modifications. The increasing number of bacteria becoming resistant to many antibiotics by horizontal gene transfer has become a major public health problem. This increase is the result of human activity which has created hotspots for horizontal gene transfer, such as wastewater systems, hospitals^47^ or intensive farming. It is also in such locations that nanoparticles of variable types can be found in appreciable amounts: for example, n-ZnO as a nutritional element for cattle^48^ or food additive, n-TiO_2_ and n-Ag, in personal care or medical products as a biocide, or in waste-water sludges as a result of industrial processes. In the future, the impact of nanoparticles, in realistic exposure conditions (e.g. without shaking) and especially nanoparticle mixtures, on the appearance of antibiotic-resistant bacteria has to be studied and taken in account.

## Methods

### Bacterial strain culture media and chemicals

The *Bacillus subtilis* strain used was the 3610 strain (wild type) (personal gift, Dr Maria Laaberki). The medium was Luria-Bertani (LB): 10 g/l tryptone, 5 g/l yeast extract and 5 g/l NaCl. Cells were grown in Erlenmeyer flasks with shaking at 200 rpm at 37 °C. Erythromycin final concentration was: 0.5 μg/ml. pJim plasmid was a personal gift from Dr. Etienne Dervyn (INRA, Micalis). The nanoparticles were from SIGMA: n-TiO_2_ (ref 700347, mixture 80:20 of anatase and rutile, 33–37 wt.% in water,<150 nm volume distribution, DLS, n-ZnO (ref 721077, 50% in water, <100 nm by DLS), and n-Ag (ref 758329, 5 wt% in ethylene glycol,<100 nm by TEM) were already purchased as soluble dispersions. Their principal features have been described elsewhere^49,50^. Biofilm agar plate preparation: The growth on soft solid agar LB medium was performed using a six well multi-well plate. Each well was filled with 7 ml of LB agar (10 g/L) containing or not a stress agent: 13 μg/ml n-TiO_2_, 17 μg/ml n-ZnO, 0.03 mM ZnSO_4,_ 10^−3^ g/l silver lactate or 10^−3^ g/l n-Ag. The multi-well plate was dried overnight (15 h) at 37 °C before being used.

### Biofilm growth conditions

A 3610 *Bacillus subtilis* culture grown overnight on liquid LB at 37 °C was diluted to A_600nm_ = 0.1 in 10 ml of fresh LB medium and incubated at 37 °C and 200 rpm until the A_600nm_ reached 0.7. 3 μl of this culture were inoculated in the middle of each well of the multi-well plates. The plates were then incubated at 30 °C for 48 h. All the cells contained in a well were recovered using a sterile plastic öse. For mass spectrometry experiments, the cells were directly put in sterile microtube and quickly froze at −20 °C. For the competence tests, the cells were resuspended in competence medium (Spizizen medium 1 × (2 g/l (NH_4_)_2_SO_4_, 14 g/l K_2_HPO_4_, 6 g/l KH_2_PO_4_, 1 g/l Sodium citrate), 3 mM MgSO_4_, 0.5% Glucose) containing 20% Glycerol to final A_600nm_ = 4 and quickly froze at −80 °C.

All experiments were performed in triplicate (three independent growth cultures) and at least two technical replicates.

Proteomic methods are described in the PRIDE repository PXD006444.

### Competence tests

Cells which were frozen as described above were quickly diluted ten times in warmed (37 °C) competence medium (1× Spizizen medium, 0.5% Glucose and 3 mM MgSO_4_). Then, 1–5 μg of plasmid DNA (pJim) was added to 100 μl of defrosted cells (corresponding to 10^8^ viable cells/ml) and the mix was incubated 1 h at 37 °C and 200 rpm. After the addition of 300 μl of LB and incubation 1h30 at 37 °C and 200 rpm, 100 μl of mix was plated on LB-Erythromycin and 100 μl of appropriate dilutions were plated on LB agar without antibiotics for CFU determination. The presence of the pJim plasmid in cells growing on LB-Erythromycin plates was assessed by PCR analysis using the RepE primers: RepE-R (gacttgaacgagtaaagccc) and RepE-F (cgctcaatcactaccaagcc). YomS primers, YomS-F (ccagcaatgtcgaagtcccaa) and YomS-R (cgcaacttccccatctgaag), were also used to determine the *Bacillus subtilis* strain identity. All experiments were performed at least in triplicate (three independent growth cultures) and with at least two technical replicates.

### Proline assay

Crude extracts were prepared as described above. Aliquots of the crude extract were used to measure the total protein quantity using the Bradford assay. To measure the free proline, proteins of the crude extracts were precipitated with 10% TCA for one hour on ice, and harvested by centrifugation (13,000 g, 5 min). Proline content was assayed in the supernatant according to^51^. Briefly, 900 μl of ninhydrine mix (0.4 ml of H_2_PO_4_ 6  M + 0.6 ml Acetic acid + 25 mg ninhydrine) was added to 100 μl of supernatant, incubated for one hour at 100 °C, and then cooled at room temperature, before 1 ml of toluene was added. The mix was vigorously mixed during 10 sec and the optical absorbance at 520 nm was measured in the aqueous phase. All experiments were performed in triplicate (three independent growth cultures) with at least two technical replicates.

### Sporulation assay

All of the cells contained in each well were recovered using a sterile plastic öse and diluted in 20 mM phosphate buffer (pH 7.4) to an OD_600_ approximately of 1 before being incubated at 80 °C for 10 min. Aliquots of appropriate dilutions taken before and after heat treatment were plated on LB plates. CFU were counted after overnight incubation at 37 °C. All experiments were performed in triplicate (three independent growth cultures) and with at least two technical replicates.

### Inductively coupled plasma atomic emission spectroscopy (ICP-AES)

Shimadzu ICP 9000 (with Mini plasma Torch in axial reading mode) was used to measure the zinc content. Standard solution for AAS (Sigma Aldrich) was used to generate the calibration curve between 10 to 500 µg/L in pure water with 1% of HNO3 (Fluka). Samples were routinely incubated in HNO3 10% ON at RT. Briefly, 500 µl of each cytosolic crude extract was incubated with 90 µl of HNO3 65% ON at RT. The sample was centrifuged at 13,000 rpm for 5 min. Before to be measured pure water was added to the supernatant extemporaneously to obtain a final volume of 7 ml. Ytterbium solution standard for AAS (Sigma Aldrich) was used as an internal standard to prevent calibration drift and fluidic perturbation. The result was express in µg/L [Zn] per mg of protein.

## Electronic supplementary material

Supplementary data 1&2
