# Author Correction: Impact of nanoparticles on the *Bacillus subtilis* (3610) competence

**DOI:** 10.1038/s41598-018-24841-x

**Published:** 2018-04-19

**Authors:** Elise Eymard-Vernain, Sylvie Luche, Thierry Rabilloud, Cécile Lelong

**Affiliations:** Université Grenoble Alpes, CNRS, CEA, BIG, CBM, 17 avenue des Martyrs, 38054 Grenoble, cedex 9 France

Correction to: *Scientific Reports* 10.1038/s41598-018-21402-0, published online 14 February 2018

The Acknowledgements section in this Article is incomplete.

“This work was funded by the CNRS, The University of Grenoble Alpes. This work is a contribution to the Labex Serenade (n° ANR-11-LABX-0064) funded by the « Investissements d’Avenir» French Government program of the French National Research Agency (ANR) through the A*MIDEX project (n° ANR-11-IDEX-0001–02).”

should read:

“This work was funded by the CNRS, The University of Grenoble Alpes. This work is a contribution to the Labex Serenade (n° ANR-11-LABX-0064) funded by the « Investissements d’Avenir» French Government program of the French National Research Agency (ANR) through the A*MIDEX project (n° ANR-11-IDEX-0001–02). Finally, Dr. Mireille Chevallet is acknowledged for their assistance in performing the ICP experiments.”

