# Supplementary material for: Impact of nanoparticles on the Bacillus subtilis (3610) competence
Source: Sci Rep. 2018 Feb 14;8:2978. doi: 10.1038/s41598-018-21402-0 (PMC5813000; doi:10.1038/s41598-018-21402-0)
Supplement: Supplementary file 1 — Supplementary data 1&2 [file 41598_2018_21402_MOESM1_ESM.doc]

SREP-17-31141B

Title

Impact of nanoparticles on the *Bacillus subtilis* (3610) competence

Authors

Elise Eymard-Vernain, Sylvie Luche, Thierry Rabilloud and Cécile Lelong

**Supplementary data 1**

Representative TEM (Transmission Electron Microscopy) Images : (a) n-TiO_2_ suspension in H_2_O and (b) n-ZnO suspension in H_2_O. The morphology of samples was observed TEM after deposition of a droplet on 200 mesh carbon lacey grids.

(a)


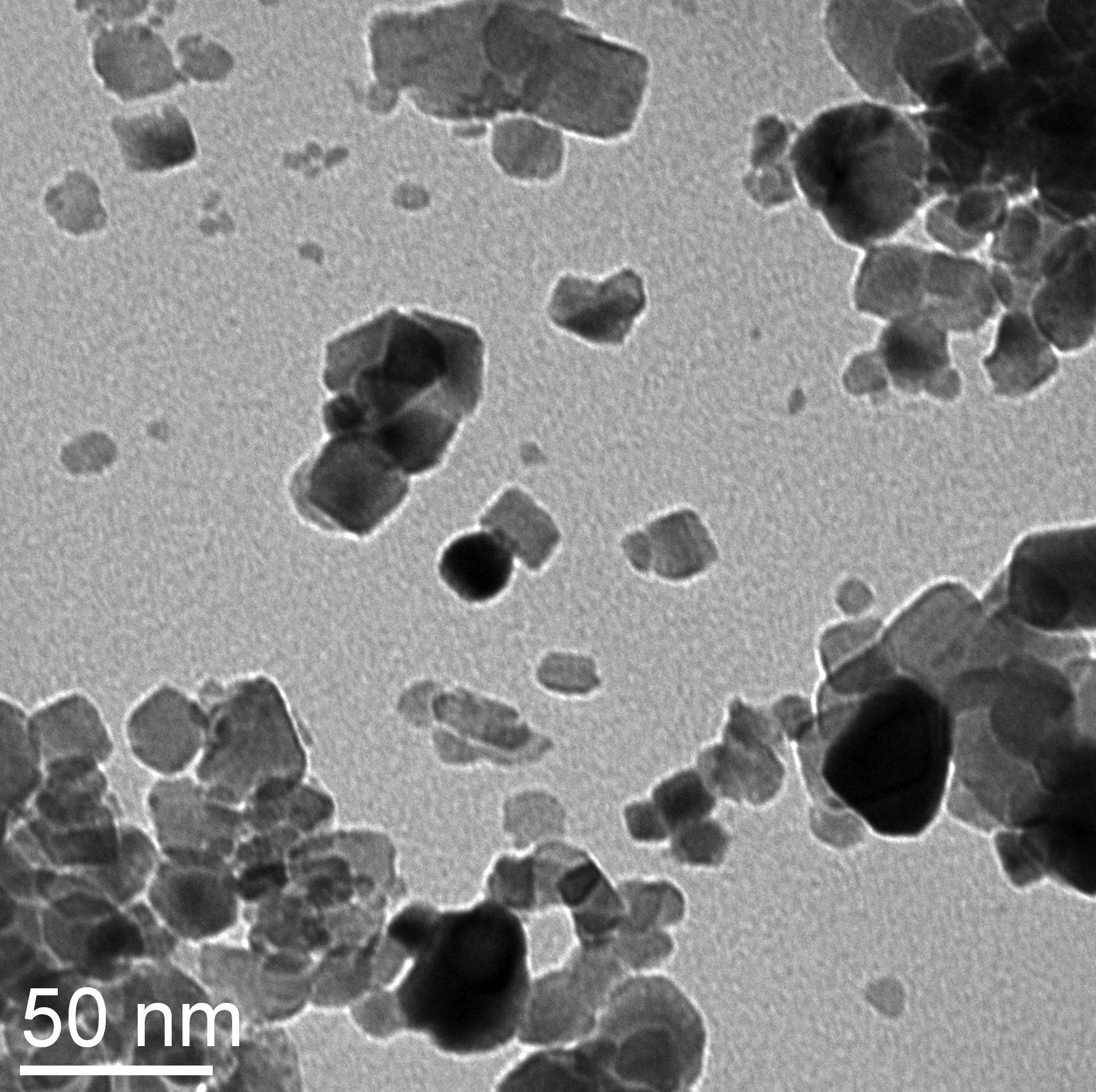


(b)


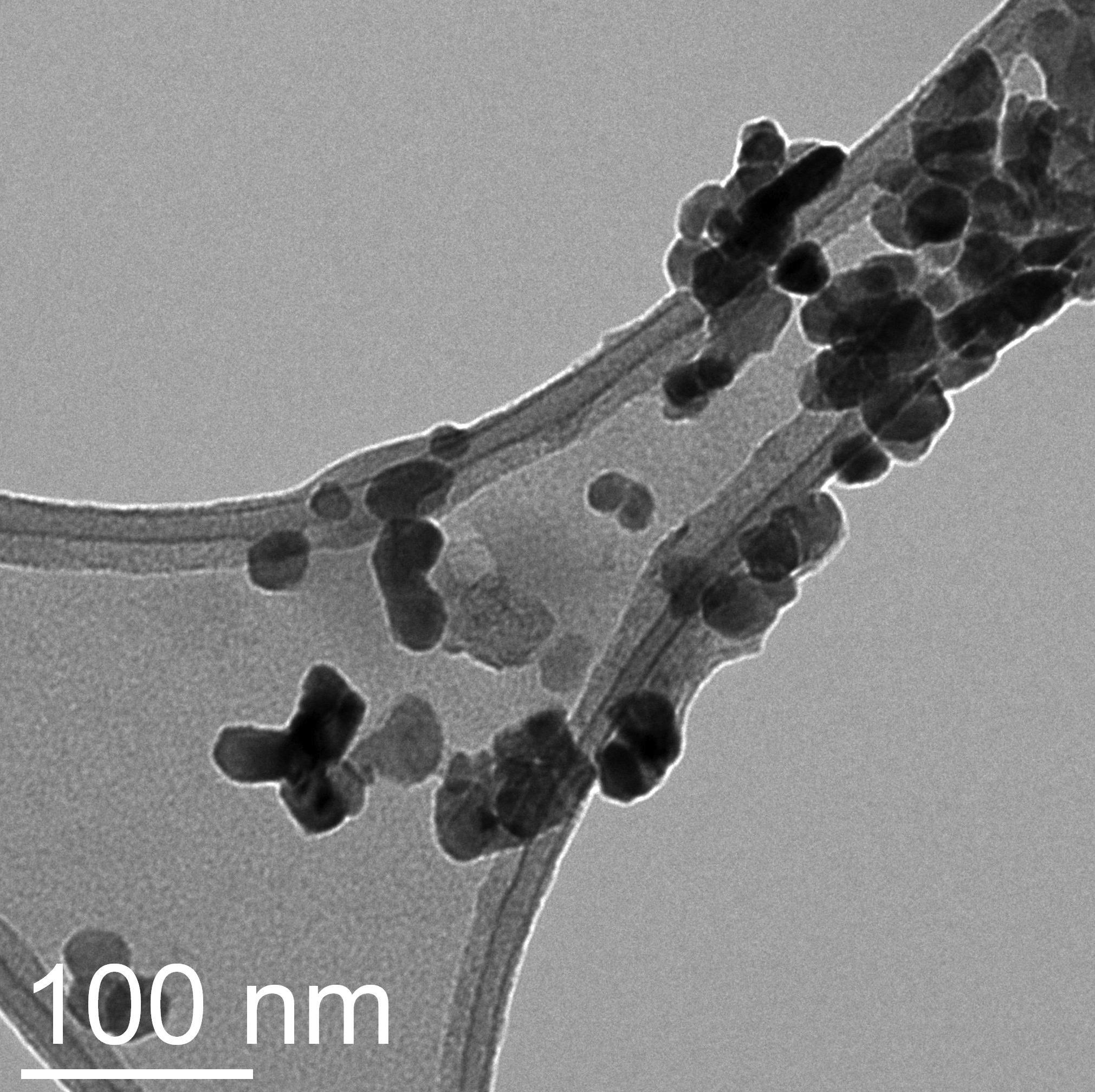


**Supplementary data 2**

Percentage of sporulation in presence of silver= (number of spore forming/CFU)x100. All experiments were performed in triplicate (three independent growth cultures) and with at least two technical replicates. All differences are significant with *p* < 0.05.
